# Small-Pox and Anti-Vaccinators

**Published:** 1881-12

**Authors:** 


### SMALL-POX AND ANTI-VACCINATORS.

The wickedness of encouraging the anti-vaccination agitation could not, it is opportunely pointed out by the *Globe*, be more strikingly proved than by an account it printed of the origin of an outbreak of small-pox in Rotherhithe. "A leading anti-vaccinator," Escott by name, who had none of his children vaccinated, has lost his wife and two children by small-pox, and four others have had the disease. Escott borrowed a suit of mourning from a friend named Angus to attend his wife's funeral, and returned the clothes without disinfection, with the result that the lender caught small-pox, and died. Since then, nearly every house in the neighborhood has been attacked, and sixteen patients have been removed to the hospital.—*Brit. Med. Jour.*

